# K-t-GRAPPA accelerated flow measurements

**DOI:** 10.1186/1532-429X-14-S1-P257

**Published:** 2012-02-01

**Authors:** Bernd A Jung, Simon Bauer, Jelena Bock, Michael Markl

**Affiliations:** 1 Dept. of Radiology, Medical Physics, University Medical Center, Freiburg, Germany; 2 Dept. of Radiology, Northwestern University, Chicago, IL, USA

## Background

Parallel imaging can reduce scan time with typical reduction factors of ~2, often not sufficient to reduce scan time to breath hold duration. Spatiotemporal parallel imaging such as k-t-GRAPPA allows for a significantly higher speed-up in data acquisition, but previous studies mostly acquired full k-space data while removing data retrospectively. Here, aortic flow scans were acquired during breath-hold using kt-GRAPPA based reconstruction [1,2] and compared to conventional protocols using GRAPPA and navigator respiration gating.

## Methods

PC imaging in the ascending aorta (venc 1.5m/s) was performed on a 3T system (Trio, Siemens) using a 12-channel thorax coil with three different scans in 10 healthy volunteers:

1) Breath-hold using kt-GRAPPA (R=5/Rnet=4.0); 2) Free-breathing using conventional GRAPPA (R=2/Rnet=1.7); 3) Free-breathing using kt-GRAPPA (R=5/Rnet=4.0).

Scan parameters were: thickness 8mm, matrix 256x160 (1.3x1.5mm), temporal resolution 28ms, 6mm navigator gating window for free-breathing scans, scan time 12s for breath-hold scan (R=5) and 55s for free-breathing scan (R=2) assuming a 50% navigator efficiency and an RR-interval of 0.9s. The reconstruction was directly implemented into the Siemens image reconstruction environment. Velocity time courses and peak velocities in the ascending aorta were determined. Further, 4D flow scans were acquired with GRAPPA(R=2/Rnet=1.6), and kt-GRAPPA (R=5/Rnet=4.4 and R=8/Rnet=6.2) in a healthy volunteer using acquisition patterns for R=5 and 8 according to [3]. Streamline visualization and velocity time courses were evaluated and compared.

## Results

**Figure 1:**
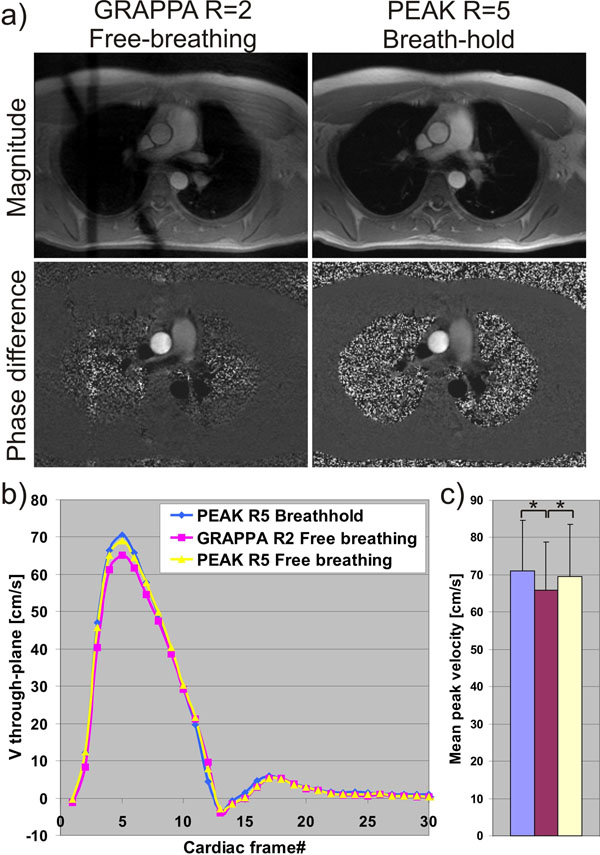


**Figure 2:**
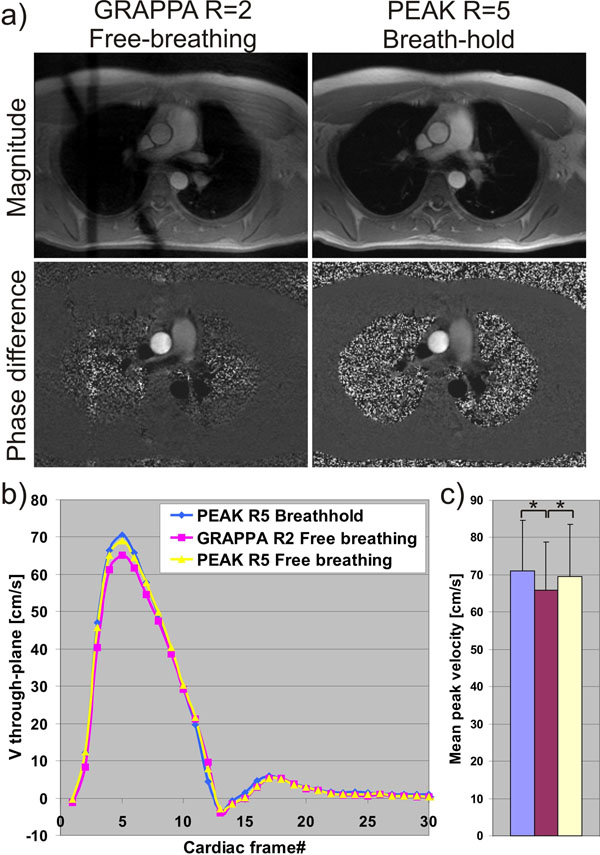


Images in Fig.1a clearly demonstrate improved image quality of the breath-hold scans compared to free-breathing. The mean velocity time courses in Fig.1b show good agreement between kt-GRAPPA and conventional 2D-PC. A slight underestimation of peak velocities by conventional GRAPPA data can be seen whereas both kt-GRAPPA scans demonstrated an excellent agreement and less low pass filtering, corroborated by the mean peak velocities in Fig.1c where GRAPPA yielded the lowest peak velocities. Streamline visualization in Fig.2a shows some noise enhancement for kt-GRAPPA (b,c) compared to GRAPPA (a) (see angiography), and a good maintenance of the velocity field distribution for R=5 as shown in the flow course in the ascending aorta (d). A slight underestimation of the velocities occurs for R=8.

## Conclusions

The results indicate that the use of accelerations factor of up to R=5 with kt-GRAPPA can provide scan time reductions that allows time-resolved 2D data acquisition during breath-hold while maintaining a high temporal resolution or 4D flow scans in ~6-8 minutes.

## Funding

BMBF 01EV0706
